# Preparation and Mechanical Properties of Alkali-Treated Wood Flour/Dynamic Polyurethane Composites

**DOI:** 10.3390/ma18163817

**Published:** 2025-08-14

**Authors:** Yifan Diao, Manyu Li, Chenglei Yu, Zhenqi Han, Shuyuan Wang, Yue Liu, Jianguo Wu, Tian Liu

**Affiliations:** 1 Key Laboratory of Bio-based Material Science & Technology, Northeast Forestry University, Ministry of Education, 26 Hexing Road, Harbin 150040, China; yifandiao2004@163.com (Y.D.); a31114528@163.com (M.L.); 18790124583@163.com (C.Y.); janet_han0219@163.com (Z.H.); wangshuyuan629@163.com (S.W.); 2 Engineering Research Center of Advanced Wooden Materials, Ministry of Education, 26 Hexing Road, Harbin 150040, China; 3 Landis Wood Co., Ltd., Pizhou 221325, China; ghzjfb@126.com

**Keywords:** wood–plastic composites, polyurethane, alkaline treatment, interface bonding, mechanical properties, reprocessing

## Abstract

In this study, alkali-treated wood flour/dynamic polyurethane composites were successfully prepared through a solvent-free one-pot method and in situ polymerization. The effects of the alkaline treatment process, changes in the flexible long-chain content in the dynamic polyurethane system, and the wood flour filling amount on the interface’s bonding, mechanical, and reprocessing properties were investigated. Partial removal of lignin and hemicellulose from the alkali-treated wood flour enhanced rigidity and improved interface bonding and mechanical strength when combined with dynamic polyurethane. The tensile strength was improved from 5.65–11.00 MPa to 13.08–23.53 MPa. As the composite matrix, dynamic polyurethane could not easily infiltrate all wood flour particles when its content was low or its fluidity was poor. Conversely, excessive content or overly high fluidity led to leakage and the formation of large pores, affecting the mechanical strength. As the polyol content increased, the matrix exhibited greater fluidity, which enabled it to accommodate more wood flour and penetrate the cell cavity or even the cell wall. This improved infiltration enhanced the interface bonding performance of the composites and made their mechanical properties sensitive to changes in wood flour content. The reprocessing ability of the prepared composites decreased with the increase in wood flour content, and the interface bonding was enhanced after reprocessing. The tensile strength retention rate of the composites prepared with alkali-treated wood flour was lower. This study provides a theoretical basis for optimizing the performance of wood fiber/dynamic polyurethane composites and an exploration path for developing self-healing and recyclable wood–plastic composites, which can be applied to building materials, automotive interiors, furniture manufacturing, and other fields.

## 1. Introduction

Wood–plastic composites (WPCs) have a wide range of applications. In WPCs, plastic serves as the matrix phase, while wood serves as the reinforcement. As a result, WPCs maximize the advantages of the two materials, featuring unique characteristics such as a light weight, good dimensional stability, corrosion resistance, durability, antibacterial properties, recyclability, low costs, and ease of processing [1]. Rapid industrialization has accelerated the global production of plastic waste, resulting in significant environmental impacts. In addition, the rising demand for wood has contributed to deforestation and increased environmental awareness [2]. To address these problems, the addition of wood residues in wood–plastic composites offers a promising solution that supports the ecological goals of “superior utilization of low-quality wood and the large-dimension use of small-diameter timber”. This approach retains the advantages of wood, including its renewability, biodegradability, low cost, and environmental friendliness [3], while reducing the use of plastic under the same circumstances. Additionally, this method can effectively reduce environmental hazards while maintaining quality of life amid ongoing social modernization. As a result, wood–plastic composites are widely used in automotive, home, construction, packaging material, electronic equipment, aerospace, and structural applications [4,5,6].

Dynamic covalent bonds have attracted considerable attention in recent years. Compared with typical polymers connected by covalent bonds—which are inert and characterized by their non-degradability, environmental insensitivity, low recovery rates, and difficulty to repair after damage—dynamic covalent bonds are adaptable and reversible. These unique properties of dynamic covalent bonds make them a promising strategy for designing an environmentally friendly wood–plastic composite matrix [7]. Introducing them into polymer materials can endow them with self-healing, shape memory properties, enhance their toughness, and induce macro-molecular structural changes. Generally, dynamic covalent bonds maintain a stable structure under normal conditions. When exposed to light, heat, or changes in pH, they undergo reversible association and dissociation through various mechanisms, such as typical ester exchange reactions, nucleophilic substitution reactions, imine bonds, acylhydrazone bonds, disulfide bonds, boric acid ester, and Diels–Alder reactions [8]. Elastomers containing dynamic covalent bonds can achieve excellent processability, mechanical properties, recyclability, and structural stability by adjusting the network synergy between dynamic bonds after forming a reversible cross-linked structure [9]. However, polymers containing only dynamic covalent bonds are sensitive to environmental changes and have low mechanical strength. Incorporating dynamic covalent bonds into other molecular networks or combining them with fillers can effectively improve mechanical properties while maintaining the original dynamic properties.

In recent years, numerous studies have been conducted on the introduction of dynamic bonds into composite materials. Cai et al. synthesized a flexible thermal ablation PDMS/PU composite elastomer with rapid room temperature self-healing properties. Its room temperature self-healing efficiency was as high as 92.9% within 3 h and could withstand flame erosion of approximately 1300 °C [10]. Nguyen et al. fabricated a dynamic covalent polymer-based carbon composite with a high dielectric constant, stable structure, and strong self-healing ability by combining a polymer with a dynamic covalent bond of disulfide with a carbon nanofiber [11]. Mo et al. used the abundant oxygen-containing polar groups of lignin to form dynamic bonds, such as hydrogen bonds and coordination bonds, in situ at the interface between lignin and polymer matrix. This strategy effectively addressed the problem of dispersion and the interface compatibility of lignin/polymer composites and improved the strength and toughness of materials [12].

Polyurethane elastomers are characterized by excellent mechanical, physical, and chemical properties and excellent biocompatibility. As a result, they are widely used in footwear, wheels, tires, machinery, sports, electronics, ships, and other applications. The introduction of dynamic covalent bonds in polyurethane elastomers can endow them with shape memory behavior, excellent electrical conductivity, and improved dielectric properties, thereby expanding their potential application range [13]. Duan et al. prepared a self-healing polyurethane elastomer by introducing a hydrogen-bond functional monomer through a Diels–Alder reaction. The elastomer exhibited a high breaking strength of 72.5 MPa, an elongation at break of 1241.0%, and a self-healing efficiency of approximately 86% [14].

Alkaline treatment can increase the internal porosity of wood material, facilitating the densification process that is used widely in business [15,16,17,18]. In addition, alkaline treatment does not alter the cellulose inside the wood lattice. However, it decreases the crystallinity of cellulose, increases the number of hydroxyl groups on the surface of wood, causes longitudinal shrinkage of the wood, and alters the bending properties of the wood [19]. In addition, the alkali gradually removes hemicellulose and lignin from wood during the treatment process. This effect becomes more pronounced with increases in the treatment temperature [20]. Moreover, alkaline treatment slightly reduces the particle size of wood flour and reduces its moisture absorption rate and maximum moisture absorption with an appropriate alkali. It also improves the mechanical properties of the resulting composites [21].

In this study, tannic acid, polyethylene glycol, and isophorone diisocyanate were used to synthesize dynamic polyurethane and prepare alkali-treated wood flour/dynamic polyurethane composites. The interface bonding of the wood flour/dynamic polyurethane composites was improved by the combined action of in situ polymerization, alkaline treatment, and hot pressing. Furthermore, the effects of flexible long chain ratios, wood flour content, and the alkaline treatment of wood flour on its mechanical properties and reprocessing properties were investigated. In the composite preparation process, the one-pot method, a solvent-free process, and simple steps were used to synthesize dynamic polyurethane without producing volatile organic compounds. The chemical structure and microstructure were analyzed using Fourier transform infrared spectroscopy and scanning electron microscopy. In addition, tensile and three-point bending tests were conducted. The tensile strength and bending strength of different samples were examined, and the change rule was discussed. This study aims to provide a theoretical basis for optimizing the performance of wood material/dynamic polyurethane composites and provides insight into the development of reprocessing wood–plastic composites.

## 2. Materials and Methods

### 2.1. Materials

Tannic acid (TA) (Aladdin, Shanghai, China), polyethylene glycol (PEG, Mn = 600) (Aladdin, Shanghai, China), isophorone diisocyanate (IPDI) (Macklin, Shanghai, China), poplar wood flour (WF) (saled, 100 mesh, Shandong, China), sodium hydroxide (NaOH) (Aladdin, Shanghai, China), sodium sulfite (Na_2_SO_3_) (Aladdin, Shanghai, China), deionized water (saled, Harbin, China).

### 2.2. Preparation of Alkali-Treated Wood Flour

First, 10 wt% NaOH and 5 wt% Na_2_SO_3_ solutions were prepared with deionized water. Then, 5 wt% wood flour (WF) was soaked in the above solution and boiled in an oil bath for 5 h. The oil bath temperature was set to 105 °C to maintain boiling during treatment. The WF was separated, washed, boiled, and repeatedly rinsed with deionized water until the pH of the filtrate became neutral. The obtained alkali-treated wood flour (AWF) was dried in an oven at 103 °C and sealed for storage in a dryer [22].

### 2.3. Preparation of a Low Transition Temperature Mixture

PEG was dehydrated under reduced pressure for 3 h in an oven at 80 °C, and TA was dried at 60 °C for 3 h. TA and PEG were mixed at molar ratios of 1:12.5 (hydroxyl ratio 1:1), 1:25 (hydroxyl ratio 1:2), 1:37.5 (hydroxyl ratio 1:3), and 1:50 (hydroxyl ratio 1:4). The mixture was heated in an oven at 80 °C to clarify the impurity-free liquid to obtain the low transition temperature mixture (LTTMs).

### 2.4. Preparation of Wood Flour/Dynamic Polyurethane Composites

The prepared LTTMs and IPDI were stirred at room temperature, and the ratio of the hydroxyl group to the isocyanate group was 1:1.05. WF and AWF were added to the mixture and mixed evenly at contents of 50, 60, 70, and 80%. They were pre-cured in an oven at 80 °C for 2 h, ground, sieved, and placed in sample bags. Then, 15 g of the pre-cured product was hot pressed in a mold (10 cm × 10 cm). It was initially heated at 3.0 MPa and 60 °C for 10 min, then heated to 100 °C, and pressed for 2 h. Finally, the pre-cured product was cooled to 20 °C for 10 min, resulting in wood flour/dynamic polyurethane composites (WFPU) and alkali-treated wood flour/dynamic polyurethane composites (AWFPU).

### 2.5. Preparation of Reprocessed Wood Flour/Dynamic Polyurethane Composites

The WFPU and AWFPU were crushed to obtain the same particle sizes, and 10 g of the particles were reprocessed in the above mold. Reprocessed wood flour/dynamic polyurethane composites (Re-WFPU) and reprocessed alkali-treated wood flour/dynamic polyurethane composites (Re-AWFPU) were prepared by hot pressing the WFPU and AWFPU particles at 100 °C under 3.5 MPa for 2 h and cooling to 20 °C for 10 min.

### 2.6. Measurement and Characterization

Fourier transform infrared spectroscopy (FTIR, Nicolet 6700, Thermo Fisher Scientific, Waltham, MA, USA) was used to characterize the composites and their raw materials, prepared with WF before and after alkaline treatment and reprocessing. The samples were scanned 32 times per minute at a resolution of 4 cm^−1^ and a scanning range of 4000–425 cm^−1^. Scanning electron microscopy (SEM, Thermo Scientific, Waltham, MA, USA) was used to analyze the cross section of composite samples prepared from WF before and after alkaline treatment and reprocessing. It was also used to analyze the microstructure and interface bonding of WF before and after alkaline treatment.

The tensile test and three-point bending test were conducted on composite samples prepared with WF before and after alkaline treatment and reprocessing using a microcomputer-controlled electronic universal testing machine (LD22.103, LabSans Material Testing Co., Ltd., Shenzhen, China) to obtain the tensile strength and bending strength. Each sample was tested more than 3 times, eliminating errors and outliers, and the average and standard deviation of the sample were calculated. The formula of the sample standard deviation is as follows:

(1) $$S=\sqrt{\frac{{\sum \left(X_{i}-\bar{X}\right)}^{2}}{n-1}}$$

## 3. Results and Discussion

### 3.1. Chemical Structure

**Figure 1:**
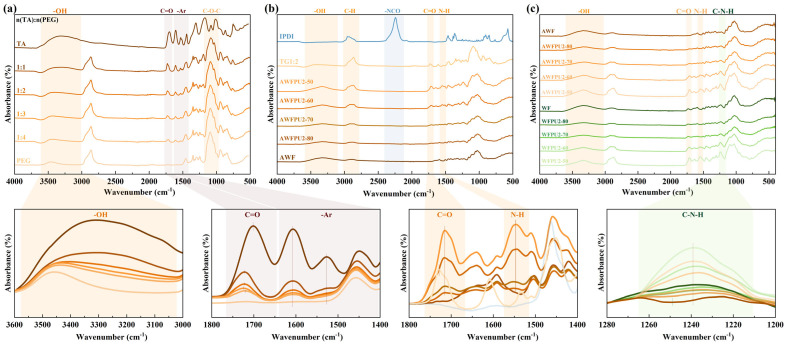
Infrared spectral images. (**a**) LTTMs and their raw materials, (**b**) AWFPU with different wood flour contents and its raw materials, (**c**) WF and AWF and their composites.

Figure 1a shows the infrared spectrum image of the prepared LTTMs and their raw materials. The peak shape of the hydroxyl peak was asymmetrically shifted, indicating a hydrogen-bond network in the PEG, TA, and prepared mixture. In addition, the C=O stretching vibration peaks (1710 cm^−1^) in the LTTMs also exhibited different levels of blue shift [23], indicating the existence of a hydrogen bond between the OH groups in the solvent and the C=O groups in the TA. Furthermore, the shift in the solvent with a ratio of 1:3 was the most obvious, exhibiting the strongest hydrogen bond [24]. The change in the ratio of TA and PEG affected the hydroxyl peak shape in the solvent, and it shifted by different degrees with the concentration, which was attributed to the coupling of the hydroxyl peak from the PEG (3460 cm^−1^) and TA (3310 cm^−1^). The stretching vibration peak of the C-O-C ether bond in the long chain of the PEG (1160 cm^−1^) [25], the characteristic peak of the skeleton vibration (1920–2250 cm^−1^) of the benzene ring of the TA, and the absorption peak of the C=O stretching vibration [23] all exhibited gradient changes in intensity with changes in the solvent concentration, instead of any other chemical reaction. In summary, different proportions of LTTMs were successfully prepared—the two solids formed a clear solution at a temperature below their melting point due to hydrogen bonding between the OH groups and the C=O groups in TA, and the effect was strongest in the 1:3 solvent.

Figure 1b shows the infrared spectral image of AWFPU and its raw materials. After mixing WF with solvent and IPDI, the carbamate bond was synthesized through in situ polymerization under controlled temperature and pressure conditions. The characteristic peak of the -NCO group (2240 cm^−1^) in the composite disappeared [26], indicating that the isocyanate group in the raw material reacted with the -OH in the LTTMs and WF, forming a carbamate bond, and was fully consumed. As the WF content increased, the C=O stretching vibration characteristic peak (1730 cm^−1^) and N-H bending vibration characteristic peak (1550 cm^−1^) of the carbamate bond gradually weakened with decreasing polyurethane content, showing a gradient change, which was consistent with expectations [27]. In addition, The shape of the OH absorption peak at 3340 cm^−1^ remained symmetrical and became smooth with increases in WF content, which indicated that the synthesis of the carbamate bond broke the hydrogen bond in the solvent, and this O-H absorption peak was coupled with the N-H bond stretching vibration peak [28]. The C=O bond (1715 cm^−1^) from the solvent red-shifted with decreases in hydrogen bonds, which is consistent with this conclusion. The stretching vibration characteristic peak shape of the C-H bond (2800–3020 cm^−1^) in the composites was affected by IPDI, LTTMs, and WF, and exhibited an intensity gradient with changes in WF content [29]. In conclusion, the carbamate bond was successfully synthesized in the AWFPU samples, and the hydrogen bond in the LTTMs was broken during this synthesis.

Figure 1c shows the infrared spectrum image of wood powder and its composites before and after alkaline treatment, to investigate the chemical structure changes of wood flour and composites caused by alkaline treatment. The C-N-H stretching vibration peak in the guaiacyl ring of WF (1250 cm^−1^) disappeared after alkaline treatment, and the C=O characteristic peak of WF (1730 cm^−1^) disappeared because of the partial removal of lignin and hemicellulose after alkaline treatment [30]. In addition, the presence of carbamate characteristic peaks at 1730 cm^−1^ and 1550 cm^−1^ confirmed that the polyurethane was successfully synthesized in these composites. As the WF content decreased, the IPDI and solvent content increased, the hydroxyl group at 3340 cm^−1^ and the ether bond characteristic peak at 1240 cm^−1^ showed a concentration gradient change that was consistent with expectations. In summary, the alkaline treatment successfully removed part of hemicellulose and lignin, and the carbamate bond was synthesized in the WFPU samples.

When polyurethane is in situ polymerized in wood flour particles, although part of the hemicellulose and lignin in alkali-treated wood flour are removed, there is basically no difference in the nucleophilic addition reaction, and both can consume isocyanate groups, break the original hydrogen bonds in the low transition temperature mixture, and successfully synthesize carbamate bonds.

### 3.2. Microstructure and Interface Bonding

**Figure 2:**
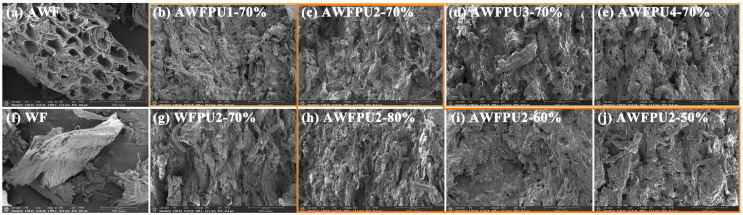
Scanning electron microscope images of wood flour and its composites before and after alkaline treatment. (**a**) AWF, (**b**–**e**) AWFPU with different contents of flexible long chain molecules, (**f**) WF, (**g**) WFPU, (**h**–**j**) AWFPU with 80%, 60%, 50% filling amount.

Figure 2 shows the SEM images of the cross-sections of WF before and after alkali treatment and the composites prepared with polyurethane. Polyurethane was uniformly deposited on WF particles through in situ polymerization. We observed that most of the damage on the cross section was caused by the fracture of polyurethane between WF particles, and a small amount of the cell wall was destroyed. The change in the amount of polyurethane outside the WF particles might be attributed to differences in the mechanical properties of the samples. Figure 2a,f compares the microscopic morphology of WF and AWF. After alkaline treatment, most of the lignin in the intercellular layer of WF particles was removed, which was consistent with the above result of the chemical structure analysis. The cell wall of wood fiber became looser and rougher. Figure 2b–e shows the composite material prepared with polyurethane of different flexible long-chain contents (1:1, 1:2, 1:3, and 1:4). As the content of flexible long chains increased, the fluidity of the system became enhanced, leading to reduced polyurethane retention on the surface of the WF particles. As a result, the polyurethane layer gradually thinned and penetrated more deeply into the WF particles. Additionally, the cell wall was gradually disrupted, and larger pores were produced. This is also supported by the increasing density of the composite samples in Figure S3 with the enhancement of fluidity. (The detailed data can be found in Table S2). Comparing the microstructure of the WFPU and AWFPU at the same wood flour content and polyurethane system (Figure 2c,g). More numerous and thicker polyurethanes were retained in untreated WF than in the treated flour. The reason was that some polyurethanes penetrated the pores generated by the AWF and remained within the WF particles, thereby reducing the amount of polyurethane on its surface. The pores generated by the polyurethane flow were fewer in number. Figure 2c,h–j compares the changes in samples with different WF filling amounts. Samples with varying wood flour contents all exhibit excellent interfacial bonding properties and demonstrate certain light transmittance (Figure S2). As the WF content increased, the cell walls were gradually disrupted, and the polyurethane between WF particles was reduced. Consequently, the pores generated by the polyurethane flow became gradually smaller, thus leading to the gradual improvement of mechanical properties. We observed that the polyurethane could not easily infiltrate all WF particles due to its low content or poor fluidity. In contrast, excessive content or high fluidity led to leakage and produced large pores. These pores caused stress concentration when subjected to external forces, thus affecting the mechanical strength. More scanning electron microscopy images can be found in Figure S1 in the Supplementary Materials.

### 3.3. Mechanical Properties

**Figure 3:**
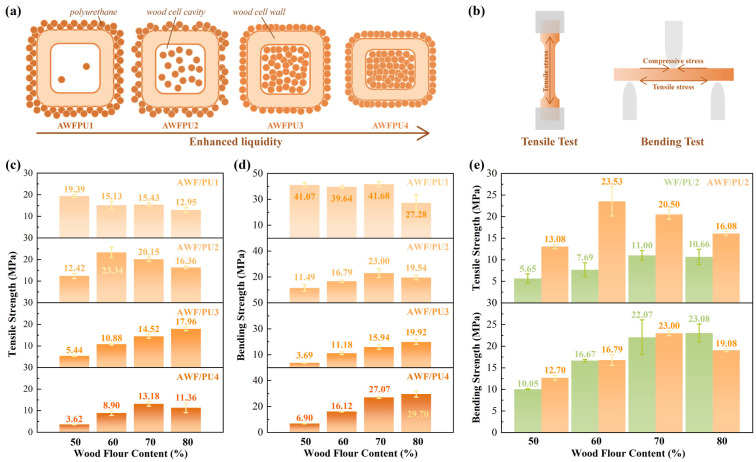
Mechanical property analysis of wood flour/dynamic polyurethane composites. (**a**) AWFPU wood cell model diagram with different content of flexible long chain molecules, (**b**) schematic diagram of tensile test and three point bending test, (**c**) tensile strength, and (**d**) bending strength of AWFPU with different filling amounts and different contents of flexible long chain molecules, (**e**) tensile strength and bending strength of WFPU and AWFPU with different filling amounts of wood flour.

Polyurethane systems containing different raw material concentrations were combined with 50–80% WF, and their tensile strength and bending strength were tested, as shown in Figure 3. The in situ polymerization technique is utilized in this process. Prior to hot pressing, the raw materials for polyurethane synthesis exist as a homogeneous liquid. Upon blending with wood flour, these liquid precursors infiltrate the pores of wood particles under applied pressure. During the hot-pressing stage, the materials remain spatially immobilized while undergoing polymerization reactions both within the wood flour pores and in the extraneous regions. This methodology facilitates the formation of continuous polymeric phases both inside and around the wood particles, thereby yielding a more robustly bonded interface. As the polyol content in the polyurethane system increased, the fluidity of polyurethane reactants improved, resulting in better wetting of the WF particles during preloading. Consequently, a greater proportion of WF could be effectively infiltrated under the same mass conditions. As the fluidity increased, more polyurethane reactants penetrated the cell cavities within the WF particles, even in the cell walls, resulting in a better interface with the WF during the in situ polymerization process. This observation was consistent with the result of the above micro-morphology analysis. However, when the fluidity was extremely high, leakage occurred during the preloading process, thereby reducing the capacity of the WF content. The wood cell wall models of different polyurethane systems are shown in Figure 3a. An increase in polyols made the synthesized polyurethane less rigid, more flexible, and more susceptible to deformation. The experimental results revealed that the weaker the rigidity of the matrix of the composite, the greater the impact of the WF content on the mechanical properties of the composite. In addition, the fractured specimen in the tensile test was subjected to uniform tensile stress. The tensile strength was mainly contributed by the polyurethane between the WF particles and the interface between the WF and the polyurethane. In contrast, the polyurethane inside the WF was trapped in the WF cell walls and did not affect the tensile strength. Because the WF particles are basically difficult to pull off, the influence in the tensile test can be ignored. In the three-point bending test, the upper surface of the specimen was subjected to compressive stress, while the lower surface was subjected to tensile stress. Therefore, the bending strength was contributed by the polyurethane between the WF particles, the interface bonding between the WF and the polyurethane, and the WF particles and the polyurethane entering the interior (Figure 3b).

As the fluidity of the system increased, the interface bonding between the WF and polyurethane increased. In contrast, the polyurethane between WF particles gradually decreased, and the tensile strength gradually decreased. When the polyurethane penetrated the interior of the WF particles and filled the cell cavity and part of the cell wall, the ability of the wood flour to resist compression and deformation was enhanced. As a result, the WF filled with polyurethane exhibited a stronger ability to resist compression. When the polyurethane system exhibited high rigidity, it did not penetrate the WF particles, indicating that the content of polyurethane between the WF was the highest, and the bending strength was the strongest (AWF/PU1). When polyurethane penetrated the WF cell cavity, the polyurethane between the WF particles decreased, and the flexural strength of the composite material gradually decreased (AWF/PU2 and AWF/PU3). The presence of polyurethane in the cell wall of the WF particles improved the interface bonding with WF and the rigidity of the WF particles, resulting in the recovery of the bending strength of composites (AWF/PU4).

Generally, as WF content increased, the mechanical properties of the wood–plastic composites initially increased and then decreased [31,32,33]. WF, a rigid filler, can resist the deformation of the material caused by external forces to a certain extent. Thus, the mechanical properties of the composite material increased with increasing WF content. However, the composite matrix could accommodate limited WF. When the WF was unable to be uniformly dispersed in the composite matrix, it agglomerated, resulting in the existence of weak phases in the material and reduced mechanical strength of the composite. This result is consistent with the results of many studies [33,34]. As shown in Figure 3c,d, the variation trend in tensile strength and bending strength of the composites with increasing WF content can be basically explained by the above findings. (The detailed data can be found in Table S1). In a system with weak fluidity, such as AWF/PU1, the polyurethane could accommodate limited WF content. Consequently, the strength decreased from 50% WF content. In the system with strong fluidity, the strength increased first and then decreased with increases in WF content. In the AWF/PU4 system, the excessive fluidity of polyurethane reactants resulted in partial leakage during preloading. As a result, some polyurethane penetrated the cell wall pores, while the content of WF that could be accommodated in the system was reduced. The results showed that the change in tensile strength of AWF/PU4 was consistent with that of AWF/PU2. During the bending test, the enhancement of the rigidity of the WF particles contributed to this consistency, maintaining the overall trend. The tensile strength and bending strength were approximately 23.34 MPa and 41.68 MPa, respectively. The performance comparison with several commercially available WPCs can be found in Table S3.

As shown in Figure 3e, the mechanical properties of the WFPU and AWFPU initially increased and then decreased, which was consistent with previous findings. The overall mechanical properties of the AWFPU were superior to those of the WFPU, and the tensile strength difference was more pronounced than the bending strength. This disparity occurred because, after alkaline treatment, the interface bonding performance between WF and dynamic polyurethane was significantly enhanced. Additionally, the content of cellulose was higher under the same conditions, and the ability of the reinforcing phase to resist fracture was stronger, while the interface was not easy to separate. Because the rigidity of the WF slightly changed, the bending resistance of the composite was slightly improved. The optimum tensile strengths of the AWFPU and WFPU were achieved at 60% and 70% wood flour content, respectively. This difference occurred because the AWF had more pores and less polyurethane between the WF particles, thereby reducing the amount of WF that the polyurethane matrix could accommodate. However, the interface bonding was more stable, consistent with the above findings.

### 3.4. Reprocessability

**Figure 4:**
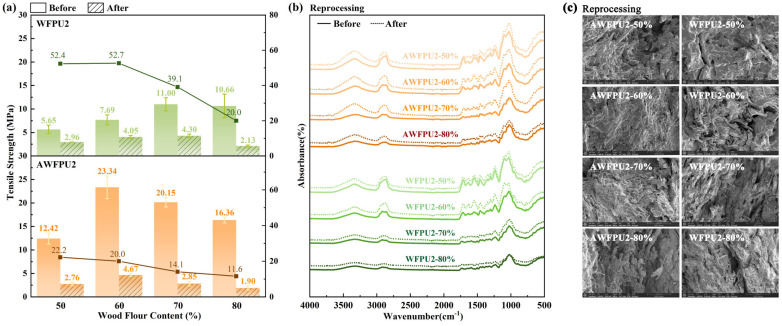
Reprocess-ability analysis of WFPU and AWFPU with different filling amounts. (**a**) tensile strength and retention rate, (**b**) infrared spectroscopic imaging, (**c**) scanning electron microscope images of the section.

The composite materials prepared with untreated and treated wood flour containing different filling amounts were subjected to hot pressing and reprocessing after mechanical crushing and then tested (Figure 4). The dynamic behavior of polyurethane within this system arises from ester exchange reactions between the phenolic hydroxyl groups in tannic acid and wood flour, and isocyanate groups. Under conditions of heating and pressure, the urethane bonds in the composite particles undergo cleavage. Through molecular thermal motion, these cleaved bonds undergo exchange with adjacent phenolic hydroxyl groups, resulting in the formation of new urethane bonds that connect the composite particles and gradually construct a three-dimensional cross-linked network structure. This macroscopic reprocessing is regulated by multiple factors, including variations in the intensity of molecular thermal motion (i.e., polyurethane flow rates), discrepancies in the exposure of phenolic hydroxyl groups before and after alkaline treatment, and the reduction in polyurethane content induced by increased wood flour filler loading. Consequently, the prepared samples exhibit distinct reprocessing properties.

As shown in Figure 4b, after reprocessing, the chemical functional groups of the composite samples slightly changed, and the hydroxyl absorption peak was slightly enhanced. This phenomenon may be attributed to the absorption of water in the environment. The ester exchange reaction occurred only during secondary processing. The tensile properties and strength retention rate (tensile strength after reprocessing/tensile strength before reprocessing × 100%) of the WFPU and AWFPU samples are shown in Figure 4a. All of the samples exhibited a certain reprocessability. As the WF content increased, the retention rate of tensile strength gradually decreased because the reprocessability of the material was mainly contributed by dynamic polyurethane. A lower polyurethane content made the ester exchange reaction less likely to occur during reprocessing. In addition, the tensile strength retention rate of the AWFPU samples was lower than that of the WFPU samples due to the presence of low polyurethane content between alkali-treated wood flour particles. These findings can be supported in the micro-topography of Figure 4c. As shown in Figure 4c, more cell wall tears were observed in the WFPU and AWFPU samples after processing than in the samples before reprocessing. This result showed that the interface bonding between WF and dynamic polyurethane was improved by reprocessing, and more fractures occurred in the cell wall, rather than in the polyurethane and interface. At the same time, fewer pores were observed in the Re-AWFPU samples, which was related to the polyurethane flow between WF particles, which was consistent with the above results and the increasing density in Figure S3 after reprocessing. Moreover, because of the more numerous pores in the AWF, the density of the AWFPU changed more after reprocessing. The decrease in tensile strength after reprocessing might be attributed to the destruction of WF particles caused by mechanical crushing, or to the destroyed ordered structure of polyurethane after reprocessing and the decreased crystallinity, or the excessive stretching of the cross-linking network caused by the absorption of water in the environment.

## 4. Conclusions

The result showed that alkali-treated wood flour/dynamic polyurethane composites exhibited excellent mechanical strength and a certain reprocessability. Alkaline treatment and reprocessing improved the interface bonding between WF and dynamic polyurethane. The partial removal of lignin and hemicellulose from the AWF resulted in a looser and rougher surface, enhancing its rigidity. This improved surface morphology contributed to better interface bonding and mechanical strength when combined with dynamic polyurethane. This improvement is attributed to the higher cellulose content in AWF at the same mass, which provides greater fracture resistance and a more stable interface. The tensile strength and bending strength of alkali-treated wood flour/dynamic polyurethane composites were higher than those of untreated composites; notably, the tensile strength was significantly improved (5.65–11.00 MPa to 13.08–23.53 MPa). In addition, the destruction of the cell wall and wood flour/dynamic polyurethane composites was mainly attributed to the fracture of polyurethane between WF particles. Dynamic polyurethane could not easily infiltrate all WF particles when its content or fluidity was poor. Conversely, excessive content or overly high fluidity led to leakage and the formation of large pores. These pores acted as stress concentrators when subjected to external forces, thus affecting the mechanical strength. As the polyol content in the polyurethane system increased, the matrix exhibited stronger fluidity, leading to improved wetting properties of WF particles. This improvement enhanced the accommodation capacity for more WF and enabled polyurethane to penetrate the cell cavity or even the cell wall, improving the interface bonding performance of the composites. As the content of flexible long-chain polyol increased, the rigidity of the composite matrix decreased, making the mechanical properties more sensitive to changes in the WF filling amount. The increase in the pores of WF after alkali treatment reduced the amount of WF that could be accommodated by the same amount of polyurethane. The tensile strength and bending strength of the treated composite were approximately 23.34 MPa and 41.68 MPa, respectively.

In the secondary processing, the material only underwent an ester exchange reaction. The reprocessing performance decreased with increases in the WF content. The tensile strength retention rate of the composites prepared with AWF (11.6–22.2%) was lower than that of the composites prepared with ordinary WF (20.0–52.4%). In addition, the secondary processing enhanced the interface bonding between WF and dynamic polyurethane, resulting in more tearing occurring in the cell wall, rather than within the polyurethane and at the interface. The decrease in tensile strength was attributable to the destruction of WF particles caused by mechanical crushing, the disruption of the ordered structure of polyurethane and decreased crystallinity after reprocessing, or the excessive stretching of the cross-linking network caused by the absorption of water in the environment. This study provides a theoretical basis for optimizing the performance of wood material/dynamic polyurethane composites and provides insight into the development of self-healing and recyclable wood–plastic composites.

## Data Availability

The raw data supporting the conclusions of this article will be made available by the authors on request.

## Supplementary Materials

The following supporting information can be downloaded at: https://www.mdpi.com/article/10.3390/ma18163817/s1, Figure S1: Microscopic morphology of all samples; Figure S2: Photos of all samples with light source at the bottom; Figure S3: Density of different composite samples; Table S1: Tensile and bending test data for all samples; Table S2: Density test data for all samples; Table S3: Performance parameter comparison of our samples and several commercially available WPCs.

## Abbreviations

The following abbreviations are used in this manuscript:

WPC	Wood plastic composite
TA	Tannic acid
PEG	Polyethylene glycol
IPDI	Isophorone diisocyanate
WF	Wood flour
AWF	Alkali-treated wood flour
LTTMs	Low transition temperature mixture
WFPU	Wood flour/dynamic polyurethane composites
AWFPU	Alkali-treated wood flour/dynamic polyurethane composites
Re-WFPU	Reprocessed wood flour/dynamic polyurethane composites
Re-AWFPU	Reprocessed alkali-treated wood flour/dynamic polyurethane composites
