# Supplementary material for: Preparation and Mechanical Properties of Alkali-Treated Wood Flour/Dynamic Polyurethane Composites
Source: Materials (Basel). 2025 Aug 14;18(16):3817. doi: 10.3390/ma18163817 (PMC12387901; doi:10.3390/ma18163817)
Supplement: Supplementary file 1 [file materials-18-03817-s001.zip › materials-3787290-supplementary.pdf]

Supplementary Materials

Preparation and Mechanical Properties of Alkali-Treated Wood Flour/Dynamic Polyurethane Composites

Yifan Diao [1,2], Manyu Li [1,2], Chenglei Yu [1,2], Zhenqi Han [1,2], Shuyuan Wang [1,2], Yue Liu [1,2,*], Jianguo Wu [3] and Tian Liu [1,2,*]

1 Key Laboratory of Bio-based Material Science & Technology, Northeast Forestry University, Ministry of Education, 26 Hexing Road, Harbin 150040, China; yifandiao2004@163.com (Y.D.); a31114528@163.com (M.L.); 18790124583@163.com (C.Y.); janet_han0219@163.com (Z.H.); wangshuyuan629@163.com (S.W.)

2 Engineering Research Center of Advanced Wooden Materials, Ministry of Education, 26 Hexing Road, Harbin 150040, China

3 Landis Wood Co., Ltd.; Jiangsu, 221325, China, ghzjfb@126.com

* Correspondence: liuyue18249010853@163.com (Y.L.); granthill63@163.com (T.L.)

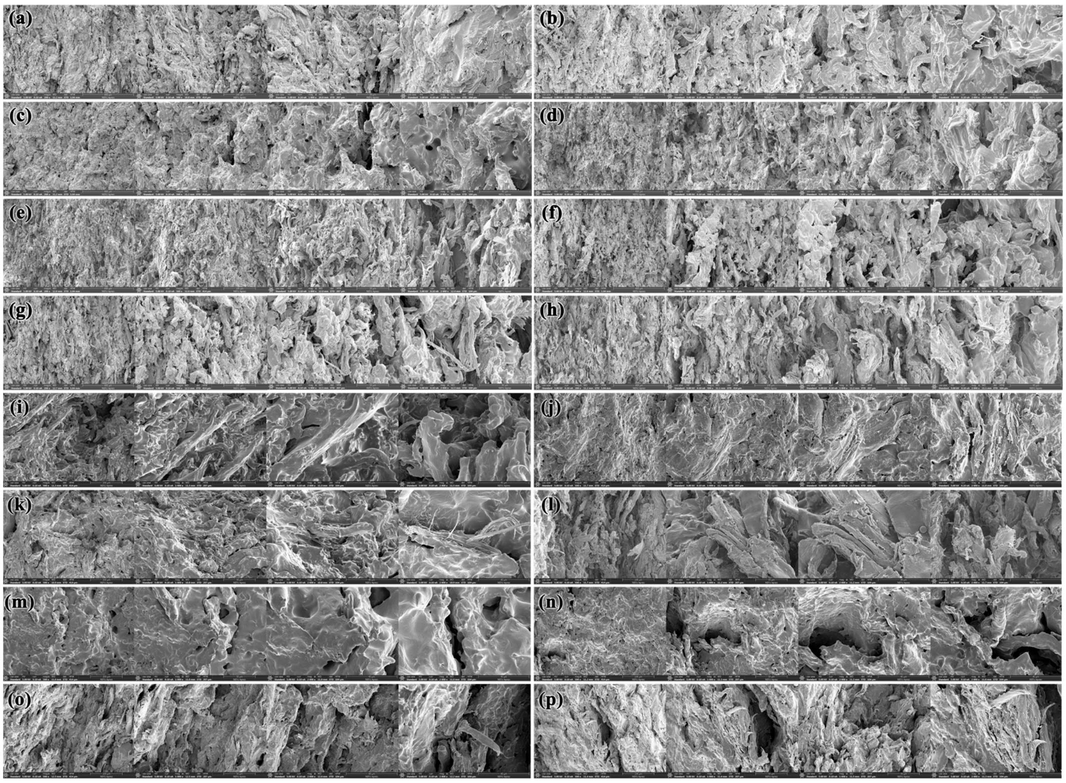

Figure S1. Microscopic morphology of all samples. (**a**) AWFPU1-70%; (**b**) AWFPU2-50%;(**c**) AWFPU2-60%; (**d**) AWFPU2-70%; (**e**) AWFPU2-80%; (**f**) AWFPU3-70%; (**g**) AWFPU4-70%; (**h**)WFPU2-70%; (**i**) Re-AWFPU2-50%; (**j**) Re-AWFPU2-60%; (**k**) Re-AWFPU2-70%; (**l**) Re-AWFPU2-80%; (**m**) Re-WFPU2-50%; (**n**) Re-WFPU2-60%; (**o**) Re-WFPU2-70%; (**p**) Re-WFPU2-80%.

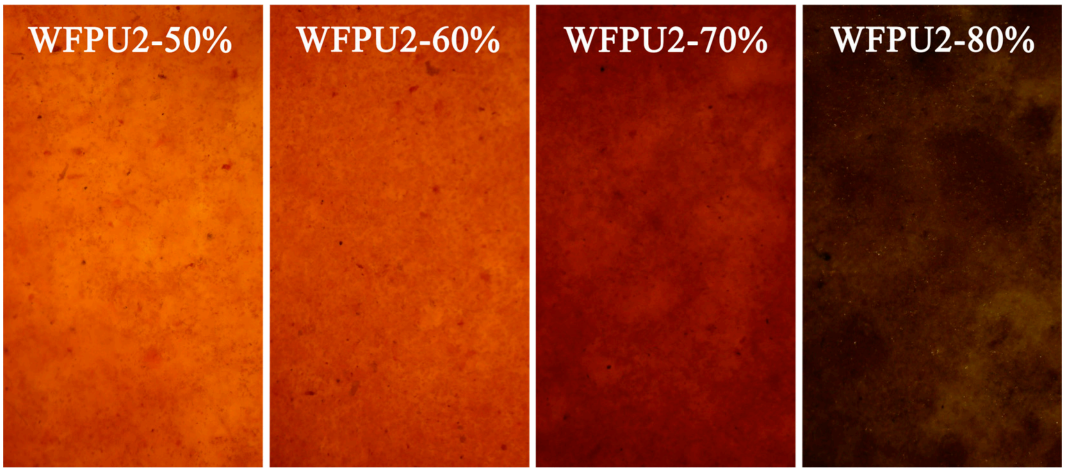

Figure S2. Photos of all samples with light source at the bottom.

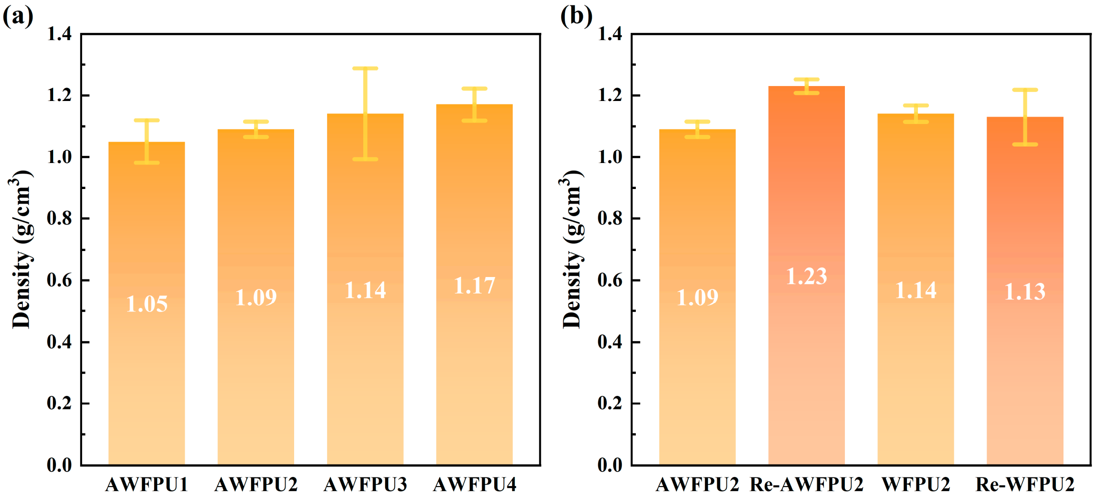

Figure S3. Density of different composite samples. (**a**) AWFPU with different flexible long chain ratios; (**b**) AWFPU and WFPU before and after reprocessing. The wood flour filling is 70%.

Table S1. Tensile and bending test data for all samples. [1]

Samples	Tensile Strength (MPa)			Bending Strength (MPa)		
AWFPU1-50%	20.150987	18.881273	19.147061	39.574852	40.956445	42.683959
AWFPU1-60%	13.265869	15.339386	16.782724	40.045767	39.865147	40.771637
AWFPU1-70%	16.263763	15.327578	14.7134	39.213541	43.645606	42.748449
AWFPU1-80%	11.590193	13.850273	13.405229	17.946544	32.464088	20.477098
AWFPU2-50%	13.43517	11.114917	12.718614	7.851646	12.76769	11.784954
AWFPU2-60%	25.926595	22.953184	21.132895	16.031478	16.54479	17.799561
AWFPU2-70%	21.302047	19.460421	19.692938	20.759852	19.310397	26.771487
AWFPU2-80%	15.725339	16.908547	16.440634	18.657962	20.788931	20.527763
AWFPU3-50%	5.673329	5.176392	5.459333	3.922178	3.413551	3.726043
AWFPU3-60%	11.281519	10.81575	10.535899	11.288556	10.369404	12.526967
AWFPU3-70%	15.531458	13.670532	14.347792	14.279037	14.580533	16.927286
AWFPU3-80%	17.387358	18.989547	17.497469	17.917662	22.294883	20.224944
AWFPU4-50%	3.516886	3.600878	3.723386	7.182868	6.684207	6.835408
AWFPU4-60%	10.815341	8.482736	8.298758	16.120857	16.404865	15.829976

AWFPU4-70%	13.094734	12.80707	13.670461	27.858374	26.862348	26.483297
AWFPU4-80%	9.394883	9.429487	11.469638	33.220693	29.974692	32.123348
WFPU2-50%	6.751161	5.271453	4.587102	11.056791	9.091688	9.132045
WFPU2-60%	6.879131	8.358625	7.487679	16.875124	16.65662	16.485154
WFPU2-70%	9.670568	13.215375	10.01089	19.449993	27.882953	17.516343
WFPU2-80%	8.244964	13.405501	12.840243	23.886452	26.254378	21.210002
Re-AWFPU2-50%	2.950710	2.589894	2.257578	—	—	—
Re-AWFPU2-60%	4.986511	4.572895	4.319874	—	—	—
Re-AWFPU2-70%	2.350387	2.996775	2.995008	—	—	—
Re-AWFPU2-80%	2.13436	2.128815	1.226237	—	—	—
Re-WFPU2-50%	2.95735	2.944032	2.982248	—	—	—
Re-WFPU2-60%	4.089148	3.768482	3.852834	—	—	—
Re-WFPU2-70%	3.968193	4.276496	4.665903	—	—	—
Re-WFPU2-80%	2.356326	2.074681	1.963475	—	—	—

[1] The tensile strength and bending strength of each sample in the table show only three data, and the amount of samples in the actual test will be more than this. More raw data of mechanical test will be made available by the authors on request.

Table S2. Density test data for all samples.

Samples	Density (g/cm^3)		
AWFPU1-70%	0.9750	1.1053	1.0789
AWFPU2-70%	1.1111	1.0909	1.0625
AWFPU3-70%	1.1000	1.3000	1.0125
AWFPU4-70%	1.2167	1.1833	1.1143
WFPU2-70%	1.1429	1.1143	1.1667
Re-AWFPU2-70%	1.2353	1.2000	1.2400
Re-WFPU2-70%	1.2333	1.0667	1.1000

Table S3. Performance parameter comparison of our samples and several commercially available WPCs [2]

Samples	Best Tensile Strength (MPa)	Best Bending Strength (MPa)	Maximum Density (g/cm^3)
Ours	23.34	41.68	1.17
PVC/WF	22	25	1.30
PP/WF	20	28	1.05
HDPE/WF	28	38	1.15

[2] PVC/WF is the product of Zhejiang Guansen New Material Co., LTD.; PP/WF is the product of Dongguan Lumeishi Environmental Protection Decoration Building Materials Co., LTD.; HDPE/WF is the product of Huangshan Huasu New Material Technology Co., LTD. The data is for reference only.
